# Identification and Expression Analysis of the *Populus trichocarpa GASA*-Gene Family

**DOI:** 10.3390/ijms23031507

**Published:** 2022-01-28

**Authors:** Kai Wu, Yanshu Qu, Hao Rong, Xin Han, Yating Tian, Li’an Xu

**Affiliations:** Co-Innovation Center for Sustainable Forestry in Southern China, Key Laboratory of Forest Genetics and Biotechnology Ministry of Education, Nanjing Forestry University, Nanjing 210037, China; wukai@njfu.edu.cn (K.W.); ysqu@njfu.edu.cn (Y.Q.); ronghao@njfu.edu.cn (H.R.); hanxin@njfu.edu.cn (X.H.); tian_yating_1014@163.com (Y.T.)

**Keywords:** *GASA*, characterization, *Populus trichocarpa*, expression profile

## Abstract

The gibberellic acid-stimulated *Arabidopsis* (*GASA*) gene family plays an important regulatory role in the growth and development of plants. In this study, we identified 19 *GASA* genes using bioinformatics-based methods in *Populus trichocarpa*, and these *PtGASA* genes could be divided into three categories based on their phylogenetic relationships. Based on an analysis of the structure and motifs of these genes, it was concluded that *PtGASA* class II members are more conserved than class I and class III members are, and the results of collinearity analysis showed that members of class II are collinearly related in poplar. Expression analysis of *Populus trichocarpa* roots, stems, and leaves showed that most of the *PtGASA* genes are expressed at higher levels in the stems or roots than in the leaves; a similar expression pattern was found in *Vitis vinifera*, indicating that the *GASA*-family members mainly play a role in the morphogenesis of poplar. Considering the phenomenon of gene amplification, we found that the higher the similarity of homologous genes was, the more similar the expression patterns. This study represents the first whole-genome identification and expression-profile analysis of the *GASA*-gene family in poplar, a model species, laying a foundation for functional studies of poplar *GASA* genes and serving as a reference for related research on other woody plant species.

## 1. Introduction

Gibberellic acid (GA) is a plant hormone that promotes growth, germination, flowering, and fruiting and affects all stages of higher plant growth [1,2,3]. The gibberellic acid-stimulated *Arabidopsis* (*GASA*) gene family is unique in plants, and the expression of its members is induced by gibberellin. In *Arabidopsis*, the gene family is referred to as *GASA* genes and has been relatively thoroughly studied [4,5,6,7,8,9,10]. GASA proteins were first identified in *Lycopersicon esculentum* in 1992 and later identified in *Petunia hybrida*, *Solanum tuberosum, Oryza sativa, Zea mays, Fragaria × ananassa* Duch, *Arabidopsis thaliana*, and *Theobroma cacao* [4,5,6,7,8,9,11,12]. *GASA* genes play vital regulatory roles in plant growth and development. They mainly participate in plant growth and development, hormone-signal transduction, and redox reactions, and these genes are involved in a series of regulatory responses, such as cell growth and division and biological and abiotic stress responses [4,13,14,15]. The proteins encoded by GASAs are small and rich in cysteine; they are mostly located on the cell wall. GASA proteins generally contain an N-terminal signal peptide and a conserved C-terminal domain. The signal peptide is composed of 18–29 amino-acid residues and functions in extracellular-protein secretion. The conserved domain is approximately 60 amino acids in length and contains 12 completely conserved cysteine residues, which is the key to maintaining the spatial structure and function of the GASA protein. A hydrophilic zone consisting of polar amino-acid residues lies between the N-terminal signal peptide and the C-terminus [9,10,16,17].

With the identification and cloning of *GASA* genes in various plant species, some of their functions such as regulating cell division, promoting flowering, and improving abiotic stress ability have been revealed [10,15,17,18]. Ben-Nissan et al. discovered *GIP* homologous *GASA* genes in *Petunia hybrida*, in which *GIP1* and *GIP2* are expressed at high levels during cell elongation, while *GIP3* and *GIP4* are expressed at high levels during cell division, ultimately inhibiting expressions of *GIP2* transgenes; these transgenic plants presented shortened internodes, the phenomenon of which is speculated to play a certain role in cell growth [5]. Zhang et al. found that members of the *Arabidopsis GASA*-gene family are mostly expressed in the flowers, stem apical meristem, and root apical meristem and speculated that this gene family may regulate cell division [10]. Based on the results of a gene chip analysis, Zhao et al. identified a gene that promoted the rapid growth of *Populus deltoides*. Because it is highly similar to *Arabidopsis GASA4*, it was named *PdGASA4*. After *PdGASA4* was overexpressed in *Arabidopsis*, it was found that *PdGASA4* promoted early bolting, leaf elongation, and stem elongation and thickening. Previous studies have shown that overexpression of beech *FsGASA5* can increase salt, oxidation, and heat-stress tolerance during seed germination and seedling growth, and overexpression of *GmGASA32* can interact with *GmCDC25* to increase the height of soybean plants [1,19,20]. In *Citrus*, *CcGASA4* interacts with two proteins (PRPBAG6-A and CNOT3) to participate in the stress response [21]. Members of the *GASA* family may exhibit opposite functions. For example, in *Arabidopsis*, *AtGASA4* promotes flowering, while *AtGASA5* inhibits flowering [14,18,22].

Poplar is the common name of Populus tree species. Poplar is the species that are widely distributed in the world, adaptable, and found mainly in northern temperate and cold regions [23]. It is also an important cultivated tree species in China. This species plays an indispensable role in timber forests (for industry) and shelter forests in northern regions and has important ecological and economic value [24,25,26,27,28]. *Populus trichocarpa* is a poplar tree species mainly distributed in western North America. The completion of the whole-genome sequencing of *Populus trichocarpa* in 2006 greatly promoted the study of forest genomics and made this species a model tree species. However, to date, there have been no reports on the structural characteristics or functions of members of the *GASA*-gene family in poplar, and there are few studies on this topic in woody plant species on the whole. The clarification of the whole genome could help us breed fast-growing and resistant trees. Therefore, in this study, the *GASA* family genes were identified from the genome of *Populus trichocarpa*, and the distribution of each gene on the chromosome, the structural characteristics of the genes, the similarities and differences with other species *GASA*-gene families, and the phylogenetic relationship were analyzed. Through bioinformatics analysis, comparison between species, combined with real-time quantitative detection of different tissues of *Populus trichocarpa*, the possible functions of some genes were speculated. The study would enrich the whole genome information of poplar, which will provide a basis for determining the function of this gene family, and can also provide a reference for related research on other woody plants.

## 2. Results and Discussion

### 2.1. Composition and Protein Characteristics of the Poplar GASA-Gene Family

**Figure 1:**
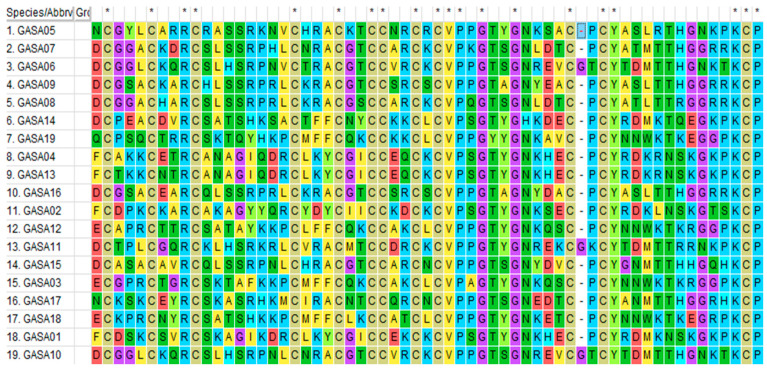
Comparison of the GASA domains from the 19 PtGASA proteins. “*” indicates a highly conserved region.

**Figure 2:**
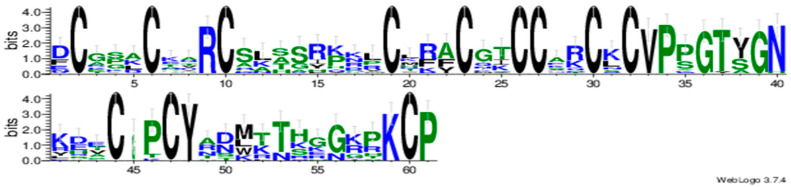
Sequence logo analysis of the conserved PtGASA domains. Each stack represented their amino acids.

We detected 19 *PtGASA* genes in the genome of *Populus trichocarpa*. Multiple sequence alignments and gene logos are shown in Figure 1 and Figure 2, and in all members of the *PtGASA* family, the C-terminus is composed of 60 amino acids (12 of which are cysteine residues), forming a conserved domain. In the multiple sequence alignment of the poplar core domain, 20 amino acids were found to be extremely conserved (amino acids 2, 6, 9, 10, 19, 23, 2, 27, 30, 32, 33, 34, 36, 39, 44, 47, 48, 59, 60, and 61 are the same in all PtGASA); 13 amino acids were extremely conserved in apple and 18 amino acids were extremely conserved in soybean (except for mutations in *GmGASA20*). The core GASA domain of poplar is more conserved than that of apple and soybean [16,29]. 

Protein is the most basic substance of life phenomena. It exists in various forms and functions in organisms, some of which are structural substances and some of which are functional substances. Therefore, it is of great significance to study the characteristics and structure of gene-family proteins for the origin and evolution of species. Therefore, we analyzed the proteins’ characteristics, the results of which are shown in Table 1. The isoelectric point of the poplar GASA proteins was between 7.96 (PtGASA03) and 9.83 (PtGASA04). The protein length was between 88 (PtGASA14) and 245 (PtGASA04) amino acids, with an average length of 110 amino acids; the PtGASA04 protein was 245 amino acids, which was quite different from that of the other proteins. This phenomenon also occurs in Arabidopsis (the AtGASA13 protein is 275 amino acids in length) and apples (the MdGASA24 protein is 305 amino acids in length) [16]. In addition, most of the PtGASA proteins were unstable. Only six were stable proteins (PtGASA02, PtGASA08, PtGASA12, PtGASA13, PtGASA15, and PtGASA16); the remaining 13 were unstable proteins; and the instability index of PtGASA04 was as high as 82.98. Studies have shown that this stability is closely related to the longevity of proteins involved in cellular enzyme reactions [11,30]. All the proteins have more positively charged amino acids than negatively charged amino acids, so the isoelectric point is almost always more than eight. The aliphatic index was between 49.27 (PtGASA04) and 83.17 (PtGASA07); most of the proteins were hydrophilic; and only six were hydrophobic, namely, PtGASA06, PtGASA07, PtGASA08, PtGASA09, PtGASA15, and PtGASA16.

### 2.2. Distribution Characteristics of Poplar GASA Genes across the Chromosomes

**Figure 3:**
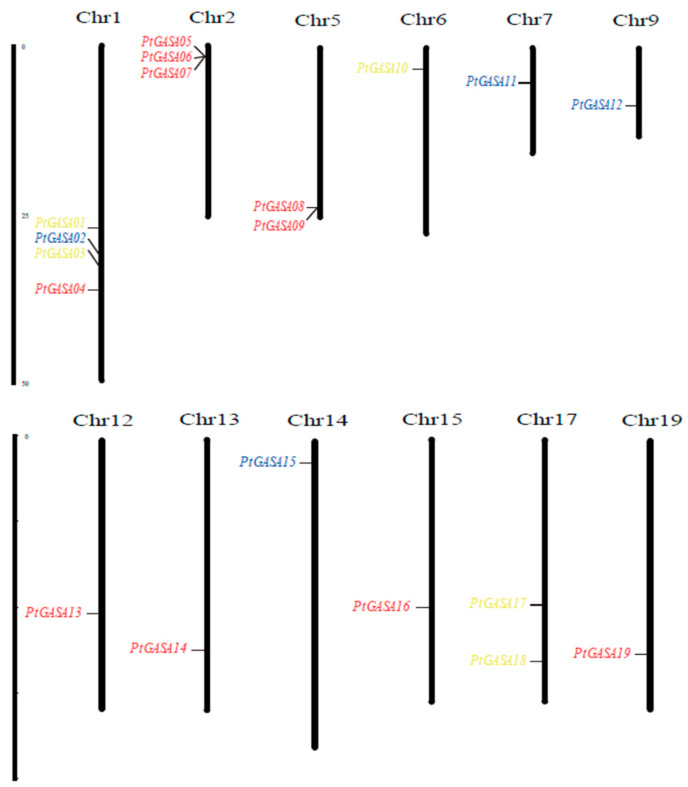
Chromosomal locations of the 19 PtGASA genes. The number of each chromosome is given above the lines. The left side of each chromosome is related to the approximate physical location of each PtGASA gene.

The 19 *GASA* genes are located on 12 chromosomes (chromosomes 1, 2, 5, 6, 7, 9, 12, 13, 14, 15, 17, and 19), and these genes were named *PtGASA01* through *PtGASA19* according to their position on the chromosome. As shown in Figure 3, poplar *GASA*-gene-family members were mainly distributed on chromosome 1 (with four genes), followed by chromosome 2 (with three genes) and chromosomes 5 and 17 (with two genes). Only one gene was found on each of the remaining eight chromosomes (6, 7, 9, 12, 13, 14, 15, and 19). The irregular distribution of *GASA* genes also occurs in apples, soybeans, potatos, grapes, and other species [12,16,29,31]. For example, in apples, 26 genes are distributed on 11 chromosomes, most of which are distributed on chromosomes 9 and 17; in soybeans, 37 genes are distributed on 15 chromosomes, most of which are on chromosomes 6 and 13 [16,29]. In addition, members of the gene family also showed the characteristics of a small number of members and a low molecular weight, which was consistent with the results of previous studies [11,16].

### 2.3. Classification of the Poplar GASA-Gene Family

**Figure 4:**
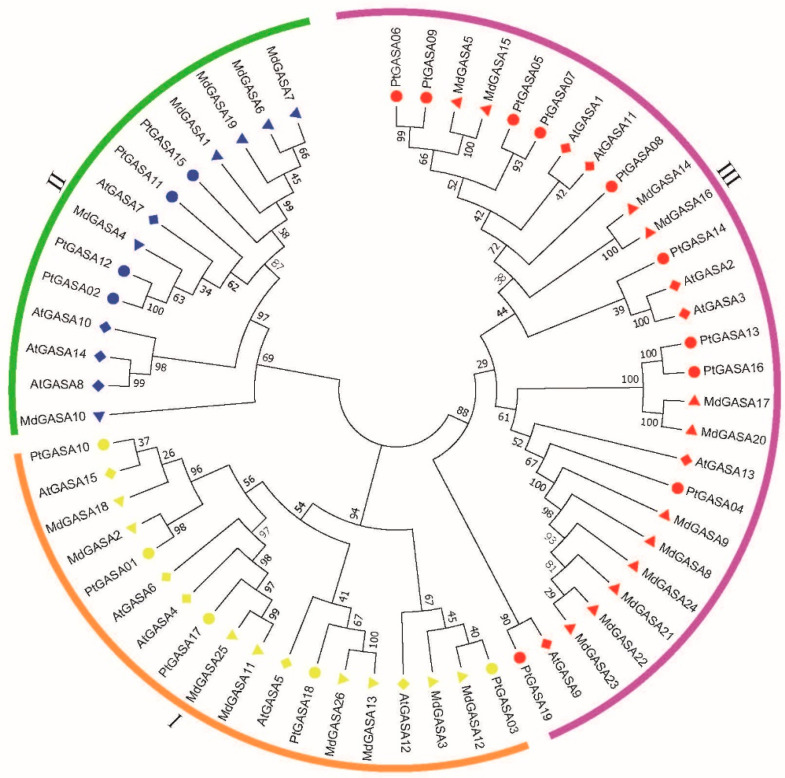
Phylogenetic tree of *GASA* genes of *Populus trichocarpa*, *Malus domestica*, and *Arabidopsis thaliana*. Circles represent poplar proteins; squares represent apple proteins; and diamonds represent *Arabidopsis* proteins. Different colours represent gene categories.

To better determine the evolutionary relationship among the members of the *PtGASA*-gene family, we constructed a phylogenetic tree (neighbor-joining [NJ] tree) comprising the sequences of 15 AtGASA proteins of *Arabidopsis* and 19 PtGASA proteins of poplar. According to the phylogenetic tree (Figure 4), the 34 GASA genes could be classified into three categories, namely, class I (16 genes), class II (8 genes), or class III (16 genes) [32]. In addition, grapes, soybeans, rice, corn, and other species were also clearly divided into three categories [33,34]. Abdullah et.al divided *TcGASA* into five categories in *Theobroma cacao*, which is essentially the same as our classification, and they divided class III into three categories [11]. In view of this, we finally decided to divide them into three categories according to the traditional method.

Within the phylogenetic tree, five genes of the members of the poplar GASA family were clustered in class I (*PtGASA01*, *PtGASA03*, *PtGASA10*, *PtGASA17*, and *PtGASA18*), and four genes were clustered in class II (*PtGASA02*, *PtGASA11*, *PtGASA12*, and *PtGASA15*). The remaining 10 genes were clustered in class III (*PtGASA04*, *PtGASA05*, *PtGASA06*, *PtGASA07*, *PtGASA08*, *PtGASA09*, *PtGASA13*, *PtGASA14*, *PtGASA16*, and *PtGASA19*).

To study the role of the *GASA* gene in poplar more accurately, we also constructed an NJ tree comprising the sequences of 19 PtGASA proteins of poplar and 26 MdGASA proteins of apples and obtained similar classification results as those above. In addition, an NJ tree was constructed for the 60 abovementioned *GASA* genes of poplar, *Arabidopsis*, and apples, and the classification results obtained were consistent with the other two phylogenetic trees.

### 2.4. Homologous Genes and Functional Prediction of the PtGASA Genes in Arabidopsis and Apples

Genes across many different species are homologous. Genes that are grouped into a class with high confidence in an evolutionary tree are likely to have the same or similar functions, and such genes are most likely homologous. BLASTP bidirectional alignment combined with the phylogenetic tree was used to predict genes homologous to *PtGASA* in *Arabidopsis* and apples (E-value = 1 × 10^−20^. Five homologous genes were ultimately found in *Arabidopsis*, and 11 homologous genes were found in apples. The specific corresponding relationships are shown in Table S1. There are more homologous genes between poplar and apples than between poplar and *Arabidopsis*. The underlying reason may be that both poplar and apples are perennial tree species, whereas *Arabidopsis* is an herbaceous species, and thus poplar and apples are more closely related.

Given that *GASA* family members have been studied in *Arabidopsis* in depth, the function of poplar *GASA* genes can be predicted via homology between *PtGASAs* and *AtGASAs*. For example, in *Arabidopsis thaliana*, *GASA5* is involved in flowering and stem growth and plays a negative regulatory role in the response to heat stress; because *PtGASA18* is homologous to *AtGASA5*, it is inferred that *PtGASA18* may have a function similar to that of *AtGASA5* [18,35]. Another example involves apple *MdGASA11/25* being homologous to poplar *PtGASA17*; *MdGASA11/25* is downregulated by GA3 and 6-benzylaminopurine (6-BA), so *PtGASA17* may also respond to this hormone regulation [16]. The remaining homologous genes can also be used for functional prediction in this way.

### 2.5. Exon–Intron Structure Analysis of PtGASA Genes

**Figure 5:**
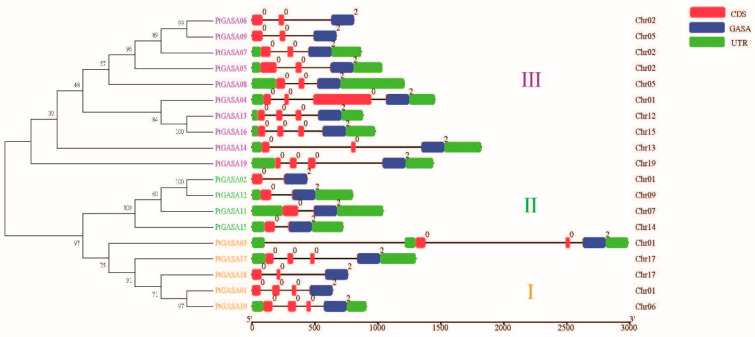
Phylogenetic relationships and gene structures of the *PtGASA* genes. Font colors represent different categories.

The study of the gene structure diversity among gene families is an important component of studying biological evolution. Therefore, an exon–intron structure map of poplar *GASA* genes was constructed through TBtools software, and each phylogenetic group was shown with different colors for clarity (Figure 5). Through the analysis of gene structure, it was found that the *GASA* family members in class I of poplar had four exons, except that *PtGASA3* and *PtGASA18* contained four exons. The two class II members were unique, both of which contained two exons and one intron. The class I and class II members were closely related. At the same time, six members of class III contained three exons, and the remaining contained four exons. These same findings were also reported in grapes and apples, indicating that the *GASA* family members of class II are highly conserved. Accordingly, class II members are more conservative than class I and class III members [16,33]. Genes with a similar number, location, and exon length are likely to perform the same or similar functions and thus may be homologous [33]. In the same class, the number of exons and the length of their positions are roughly the same, which also corresponds to the results of our phylogenetic tree. Even in the same class, the distribution of introns’ length was different and was more variable compared with that of exons because introns are not involved in coding. Of course, there are also individual differences. For example, the length of one exon of *PtGASA4* is significantly longer than that of other exons of this gene family and even longer than that of its homolog (*PtGASA16*). This phenomenon also occurs in other gene families. Xu et al. investigated the structural differences during the evolution of 612 pairs of sibling homologous genes from seven representative gene families and found that the exon–intron structural differences of homologous genes were caused by three mechanisms: exon replication/intron loss, exon/pseudoexon transformation, and insertions/deletions [31].

### 2.6. Analysis of GASA Gene Amplification and Conserved Motifs in Poplar

Duplicate genes are among the sources of the biological evolution of gene function [36]. In the search for homologous poplar genes, it was found that the three genes, i.e., *PtGASA5*, *PtGASA6*, and *PtGASA7*, are tandem repeats, and the remaining genes are scattered repeats (Table 2). These findings indicate that the poplar *GASA* genes evolved as a result of tandem repeat events and scattered repeats, with the former having a greater impact. The cause of these findings may be a whole-genome duplication event [37].

To better analyze the sequence characteristics and structural differences of the members of the poplar *GASA*-gene family, MEME software was used to predict the protein sequences of the members of this family. Motifs generally have sequence-specific protein-binding sites (for functioning as transcription factors) or are involved in important biological processes (RNA initiation, termination, shearing, etc.) and are generally considered to have biological importance [12]. We predicted a total of 10 conserved motifs among the members of the poplar *GASA*-gene family; these motifs were named motif 1 to motif 10. Although there were differences in the length and the number of motifs across the GASA-protein sequences, the distribution of motifs within the same group was similar.

**Figure 6:**
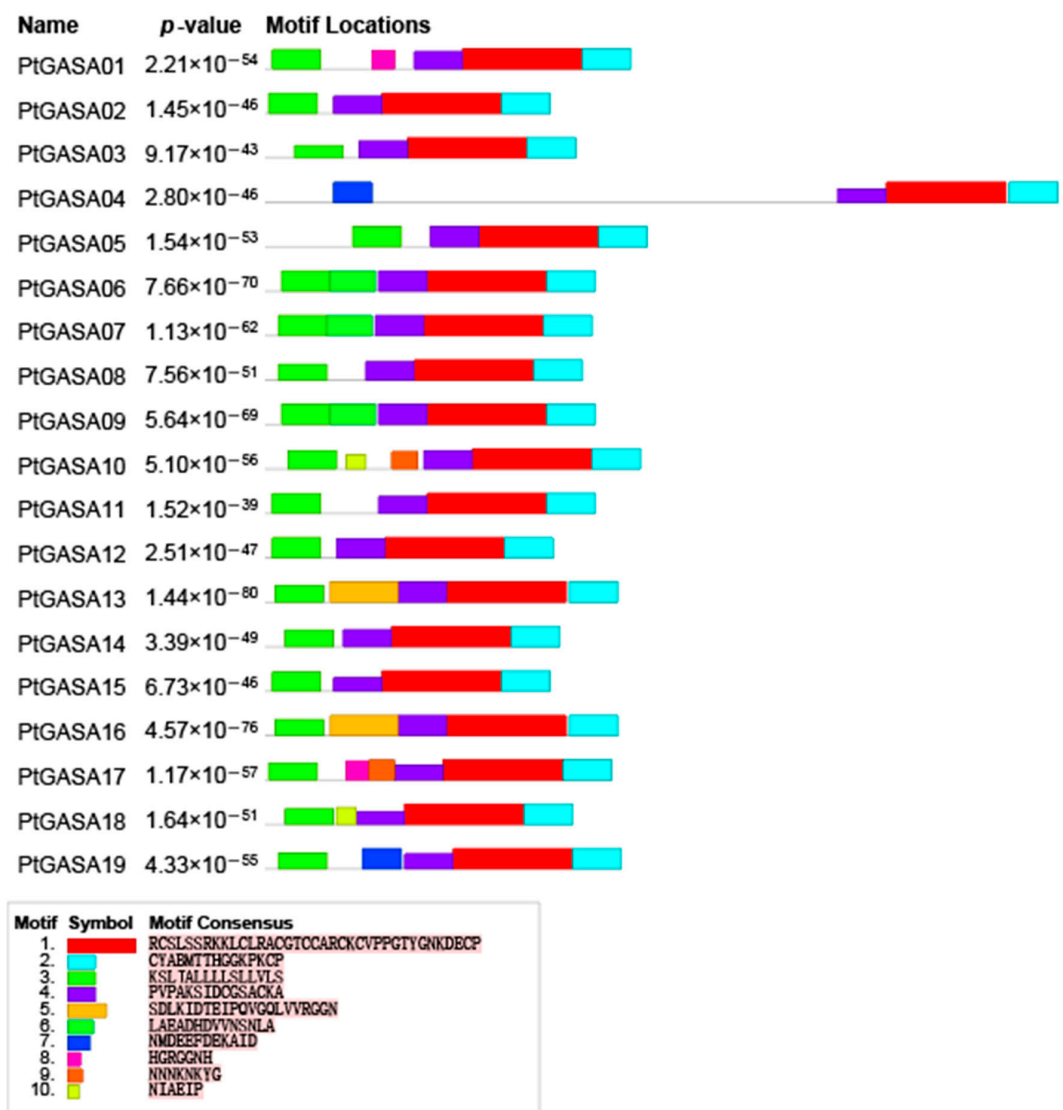
Motif analysis. The different colors of boxes denote different motif numbers. The length of box indicates motif length.

Figure 6 shows that class I members contain four to six motifs, class II members contain only four motifs, and class III members contain four to five motifs. These results provide strong evidence that the class II members of this family are more conserved than other class members are. Motif 1, motif 2, and motif 4 are present in all the *PtGASA* genes. This was verified by SMART, and it was found that these three motifs constitute the GASA domain. During the verification process, it was found that this gene family was extremely conserved. In the SMART analysis of the 19 protein sequences, no domains other than the GASA domain were found. Motif 3 is shared by all members except *PtGASA4*. After SMART verification, it was found that this motif is located in the transmembrane helix region and represents a signal peptide. Interestingly, although SMART revealed that this motif in *PtGASA4* represents a signal peptide, this motif was not found by MEME. This is probably due to the different algorithms used by MEME and SMART; a similar phenomenon was reported in a study of *Salix suchowensis* [37]. In addition, some motifs were detected specifically in homologous genes with high similarity. For example, motif 5 is present only in *PtGASA13* and *PtGASA16* (which are 91.74% similar); motif 10 is present only in *PtGASA10* and *PtGASA18*; and motif 4 is present only in *PtGASA6/7/9*; this phenomenon has also been described in grapes [33].

### 2.7. Spatial Structure and Subcellular Localization of Poplar GASA-Gene Family Proteins

**Figure 7:**
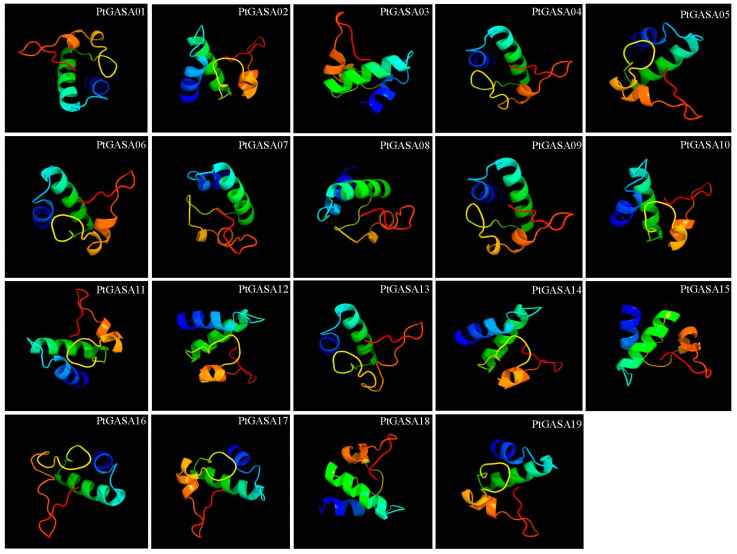
Predicted 3-D structures of PtGASA proteins.

The properties and biological functions of proteins are based on their chemical composition and structure. Subcellular localization of a protein and its tertiary structure can provide clues for exploring the role of the protein. The secondary and tertiary spatial structures of *PtGASA* family members were predicted, and it was found that the protein structure of the gene-family members was mainly composed of random coils and α-helices, but there were also a very small number of β-folds (Figure 7). Due to the presence of random coils, all members of this family have flexible structures [33]. The subcellular-localization results showed that the proteins encoded by the members of poplar *GASA*-family genes are located in four locations: the Golgi apparatus, the cell wall, the cell membrane, and the nucleus (Table S3). This is similar to the results of previous studies but not completely consistent. For example, in petunia, Western blotting proved that members of this family are located in the cell wall and the endoplasmic reticulum [5]. In rice, OsGSR1 was proven via GFP to be localized in the cell membrane, the cytoplasm, and the nucleus [8]. In *Arabidopsis thaliana*, transient transfection and transgenic methods proved that AtGASA5 was located in the cell wall and the extracellular matrix [18], while members of the poplar *GASA* family were found to be localized on the Golgi apparatus. These prediction results need to be further confirmed by subsequent experiments. Analysis of the transmembrane region of this family of proteins revealed that some of its members have a typical transmembrane α-helical region, indicating that these members may be membrane proteins and thus may participate in plant transmembrane transport, which coincides with the subcellular localization results (Figure S1).

### 2.8. Analysis of Cis-Acting Elements in Poplar GASA Promoters

**Figure 8:**
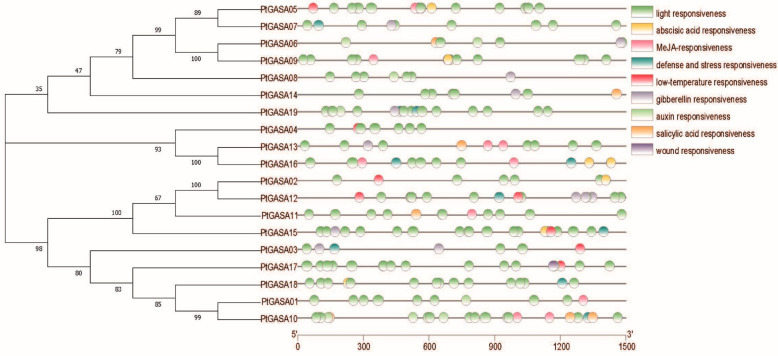
Cis-element prediction in the *PtGASA* promoters.

As DNA sequences that can affect gene expression, cis-acting elements participate in gene-expression regulation and are indispensable for further understanding the function of *GASA*-gene-family members. Various response elements were identified; these elements are specific DNA sequences that can recognize and bind to specific transcription factors, regulating the expression of specific genes. These regulatory elements represent the focus of our research on gene functions and traits, and the element prediction results are shown in Figure 8. The *GASA*-gene-family members mainly respond to light, followed by gibberellin. The promoters of the *GASA* members also contain hormone-response elements related to plant growth and development; these include elements that respond to abscisic acid, auxin, methyl jasmonate, and salicylic acid as well as low temperatures, defense, and stress. Interestingly, only *PtGASA17* was found to be a wound-response element, but this element has not been reported in apples, soybeans, grapes, and other species, indicating that *PtGASA17* may participate in the self-healing of poplar and improved vitality, although this needs to be further verified by experiments. The predicted motifs suggest that the *PtGASA* genes are likely regulated by corresponding cis-acting elements in their promoter. Therefore, these findings can promote the study of interactions that occur between cis-acting elements and genes in poplar, and these findings help clarify the hormone-regulatory network of poplar and can help in the study of the specific regulatory methods.

### 2.9. Poplar GASA Gene Evolution and Collinearity

**Figure 9:**
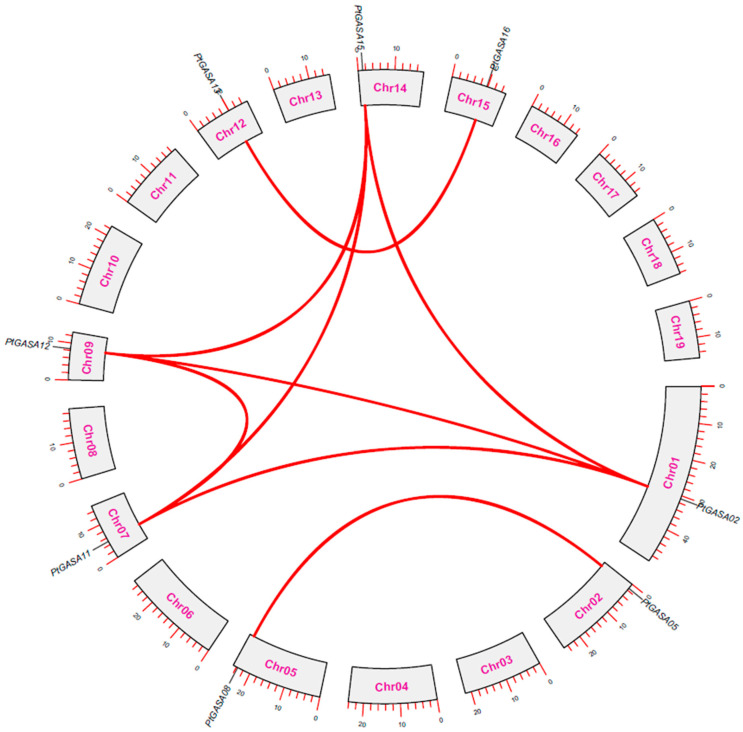
Chromosomal distribution and synteny analysis of *Populus trichocarpa GASA* genes.

DNA duplication (whole-genome duplication, tandem duplication events, fragment duplication events, etc.) cause parts of or whole chromosomes to double and can improve biological adaptability; this adaptability is one of the driving forces leading to the evolution of gene functions in species [38,39]. As shown in Figure 9, 8 genes out of 19 genes were identified in this study that may have undergone gene duplication events during evolution (*PtGASA02*, *PtGASA05*, *PtGASA08*, *PtGASA11*, *PtGASA12*, *PtGASA13*, *PtGASA15*, and *PtGASA16*). In addition to *PtGASA05*-*PtGASA08*, all of which are on chromosomes 2 and 5, and *PtGASA13*-*PtGASA16*, all of which are on chromosomes 12 and 15, four genes (*PtGASA02*, *PtGASA11*, *PtGASA12*, and *PtGASA15*) have collinear pairs. Interestingly, all these genes are class II members. In apples and grapes, class II genes also have collinearity relationships, but in poplar, all class II members have collinearity [16,33]. Collinearity is disrupted by the transposition of genes and the insertion, deletion, duplication, rearrangement, and turnover of chromosome fragments, which indicates that the duplicated regions occurring between these four genes are more conserved than the remaining two pairs of genes are [40,41].

### 2.10. Expression Analysis of PtGASA Genes in Different Tissues of Populus Trichocarpa

**Figure 10:**
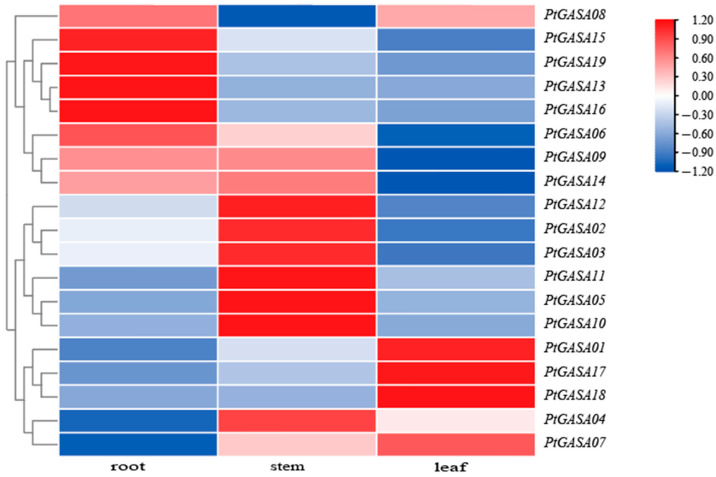
Expression profiles of *PtGASA* genes in different tissues as determined by qRT-PCR.

At present, the characteristics and expression patterns of *GASA* genes in different tissues in apples and *Arabidopsis thaliana* are relatively clear, but the expression of *Populus trichocarpa GASA* genes in different tissues is still unclear. To further analyze the potential *PtGASA* functions related to *Populus trichocarpa* growth and development, the expression level of 19 PtGASA genes in roots, stems, and leaves of Populus trichocarpa were verified by qRT-PCR (Figure 10). There are four genes (*PtGASA15/19/13/16*), six genes (*PtGASA12/02/ 03/11/05/10*), and three genes (*PtGASA01/17/18*) with the highest expression levels in roots, stems, and leaves, respectively. These genes had low expression or no expression in the other two tissues. *PtGASA6/9/14* had high expression in two tissues (roots and stems), respectively, and had no expression in leaves. *PtGASA08* was highly expressed in roots and leaves but had low expression in stems. On the contrary, *PtGASA04* was highly expressed in stems and had low or even no expression in the remaining two tissues (roots and leaves). As a comparison, *PtGASA07* was highly expressed in leaves. Therefore, the phylogenetic grouping of the *GASA*-gene family does not obviously correlate with the function of *GASA*; this phenomenon also appears in the identification of the Cacao *GASA* family [11]. However, Nawaz et al. found in the *OsCNGC*-gene family identification that genes classified as a branch of the phylogenetic tree had similar functions [42]. In general, most of the members of the poplar *GASA* family were highly expressed in the stems or roots. It can be concluded that the *GASA* family mainly plays a role in the morphogenesis of poplar, regulating stem elongation and thickening [37]. The *GASA*-gene-expression pattern in poplar leaves was different from that in grape and apple leaves. In the study of the grape *GASA* family, most members were highly expressed in the stems, leaves, and tendrils and expressed at low levels or not at all in the flowers and fruits. In apples, most *MdGASA* was highly expressed in the leaves and buds but expressed at low levels in the fruits [32,33]. In addition, we further analyzed the functional pattern of individual gene expression between tissues and found that the expression of individual genes varied greatly (Figure S2). For example, the expression of *PtGASA5/10* in stems was more than 20 times higher than that in leaves and roots; the expression level of *PtGASA18* in leaves was more than 20 times higher than that in roots and stems; and the expression level of *PtGASA13* in roots was 9 times higher than that in roots and leaves. The high expression of these genes in various tissues suggests that they may synthesize tissue specificity proteins involved in cell differentiation. In poplar, the higher the similarity of homologous genes is, the more similar the expression patterns. For example, *PtGASA02*-*PtGASA12* (96.55% similarity) and *PtGASA13*-*PtGASA16* (91.74% similarity) were highly similar, and their expression patterns in the roots, stems, and leaves were almost the same. Although *PtGASA05*-*PtGASA09* are homologous, their similarity is only 65%. The expression levels of these two genes in the stems were similar, but *PtGASA09* was highly expressed in the roots, while *PtGASA05* was not expressed in the roots. 

## 3. Conclusions

Through bioinformatics analysis, we identified 19 members of the *Populus trichocarpa GASA*-gene family. Phylogenetic analysis revealed that *GASA* members can be divided into three categories. In addition, a specific wound-response element was found in *PtGASA17*; perhaps the gene could be a candidate for breeding stress-resistant poplars. Analysis of *GASA* gene expression in different tissues of *Populus trichocarpa* showed more highly expressed genes in stems or roots than in leaves. Overall, the research results in this article lay a foundation for the identification of poplar *GASA*-gene function and identification of the structure and function of the *GASA*-gene family members, the findings of which can provide a reference for the study of the *GASA* family in other species. In addition, this study is a summary of the bioinformatics analysis and prediction-detection results by real-time quantitative for the *GASA*-gene family of *Populus trichocarpa*. The true functions of family genes still need to be verified by experiments such as overexpression, gene knockout, etc. The relationship between these genes and the final phenotype also involves various regulatory factors, which need to be further studied.

## 4. Materials and Methods

### 4.1. Plant Materials

In this study, nine tissue-culture seedlings of *Populus trichocarpa* with good growth conditions obtained from Nanjing Forestry University were used as materials. We cut 2 cm shoot tips from the existing *Populus trichocarpa* as explants. The explants were subcultured on an untreated MS medium, rooted one week later, and then continued to grow on an untreated MS medium for one month. The tissue-culture seedlings were placed in a greenhouse with a light intensity of 1000–4000 lux and a temperature of 25 °C, with 16 h of light and 8 h of darkness every day. Nine tissue-culture seedlings without pests and diseases were selected, with three biological duplications. Finally, fresh roots, stems, and leaves of *Populus trichocarpa* were removed separately, frozen immediately in liquid nitrogen, and then stored at −80 °C for RNA extraction.

### 4.2. Experimental Methods

#### 4.2.1. Data and Sequence Retrieval

The poplar genome data, including the latest version of the poplar whole-genome protein file and protein-annotation file (gff3), were downloaded from the Phytozome plant genome database (https://phytozome.jgi.doe.gov/pz/portal.html (accessed on 19 May 2021)) [43]. The sequence data of the apple GASA proteins were obtained from the Phytozome-plant genome database according to the serial number reported by Fan et al. [32]. Information on *Arabidopsis GASA* family members was obtained from the Phytozome-plant genome database and the UniProt database (https://www.uniprot.org/ (accessed on 19 May 2021)) [44].

#### 4.2.2. Identification and Distribution Characteristics of Poplar GASA Genes

First, GASA HMM files were obtained from the Pfam (https://pfam.xfam.org/ (accessed on 19 May 2021)) online website by using the name of the *GASA* family. Moreover, HMMsearch software for HMMER assembly was used to search for possible *GASA* family members within the poplar protein file. At the same time, to more accurately identify the members of the *GASA* family of poplar, BLASTP was used to compare the FASTA file of the GASA domain downloaded from the Pfam website with the full protein sequence of poplar to identify possible gene members (E-value = 1 × 10^−3^, and the intersection of the two was used to identify the potential members of the poplar *GASA* family [37]. Moreover, to ensure the reliability of all these candidate proteins, the sequences of potential gene family members identified were submitted to the SMART (http://smart.embl-heidelberg.de/ (accessed on 24 May 2021)) website and the Conserved Domains Database (CDD) of the NCBI (https://www.ncbi.nlm.nih.gov/ (accessed on 24 May 2021)) website, and any incomplete sequences of the GASA domain were removed. Moreover, the intersection of the two was used to identify the final *GASA* family members of poplar [45,46]. Then, MapInspect software was used to map the location of the *GASA* genes on the poplar chromosomes, and the genes were named according to their position on the chromosomes. Finally, the ExPASy tool was used to statistically analyze the predicted molecular weight, length, isoelectric point, and hydrophilicity of the proteins whose sequence was obtained [37].

#### 4.2.3. Multiple Sequence Alignment and Evolutionary Analysis of Poplar GASA Genes

Logos of the conserved domains of the poplar *GASA* genes was created using the web tool WebLogo 3 (weblogo.threeplusone.com (accessed on 14 September 2021)). ClustalW was used for multiple sequence alignment of the protein sequences of the identified poplar and *Arabidopsis GASA*-gene family members. MEGA 7.0 software was subsequently used to construct a phylogenetic tree of the poplar and *Arabidopsis GASA* gene families. The specific method used was the NJ method, and the parameters were set to the default values. Then, an NJ tree was constructed for all *Arabidopsis* GASA protein sequences to verify the credibility of the previous phylogenetic tree. At the same time, a phylogenetic tree comprising poplar and apple GASA protein sequences was constructed, and the method used was the same as that used above.

#### 4.2.4. Analysis of the Structure of Poplar *GASA* Genes

Structural information, such as the number of introns and exons, of poplar *GASA*-gene family members was obtained from the poplar protein annotation file retrieved from the Phytozome-plant genome database. The gene structure was determined based on the corresponding sequence, and the gene structure diagram was generated by TBtools software [47].

The online software MEME Suite 5.3.3 (https://meme-suite.org/meme/ (accessed on 5 October 2021)) was used to predict the conserved motifs. The parameter settings included the highest motif number possible (i.e., 10), and the rest were set to the default values.

To determine the similarity between poplar *GASA* genes, multiple sequence alignments and BLASTP alignments (E-value = 1 × 10^−20^ were performed. To better predict gene amplification, the following indicators were used: (1) for long genes, the proportion of regions used for comparison was ≥65% and (2) the similarity of the regions used for comparison was ≥65% [37,48].

#### 4.2.5. Spatial Structure and Subcellular Localization of Poplar GASA Proteins

The SCRATCH Protein Predictor online website (http://swissmodel.expasy.org/interactive (accessed on 18 July 2021)) was used to predict the secondary and tertiary structures of the proteins encoded by these gene family members. At the same time, the online website Plant-mPLoc (http://www.csbio.sjtu.edu.cn/bioinf/plant-multi/ (accessed on 25 July 2021)) was used to determine the subcellular localization of the poplar *GASA*-family protein members. In addition, the online software PlantCARE (http://bioinformatics.psb.ugent.be/webtools/plantcare/html/ (accessed on 13 September 2021)) was used to detect the presence of cis-acting elements in the region 1500 bp upstream of the start codon of the poplar GASA genes. Last, the online software TMHMM Server 2.0 (http://www.cbs.dtu.dk/services/TMHMM/ (accessed on 16 September 2021)) was used to analyze the transmembrane regions of the proteins [32].

#### 4.2.6. Expression Analysis of Poplar GASA-Family Genes in Different Tissues

An RNAprep Pure Plant Plus Kit was used to extract RNA from the roots, stems, and leaves of poplar plants and determine its purity and concentration. A 1.2% agarose gel (in conjunction with electrophoresis) was used to determine RNA integrity, after which reverse transcription and real-time quantitative PCR were performed, and RT-PCR primers are placed in Table S2. According to the 2^−ΔΔCT^ method, the relative expression levels of the members of the poplar *GASA*-gene family in the roots, stems, and leaves were calculated via log_2_ normalization, and then TBtools software was used to construct the expression profiles of 19 *PtGASA* genes [37,49].

## Figures and Tables

**Table 1:** Amino-acid composition and physiochemical characteristics of PtGASA proteins.

Gene ID	Sequence ID	Length (aa)	*M*_W_ (kDa)	PI	Instability Index	GRAVY	Aliphatic Index
*PtGASA01*	Potri.001G254100.2.p	113	12.26	9.48	43.90	−0.250	60.53
*PtGASA02*	Potri.001G297700.1.p	88	9.80	9.12	37.23	−0.203	56.59
*PtGASA03*	Potri.001G315500.1.p	96	10.33	7.96	52.40	−0.014	61.04
*PtGASA04*	Potri.001G350600.3.p	245	25.85	9.83	82.98	−0.477	49.27
*PtGASA05*	Potri.002G022500.1p	118	12.36	9.20	43.25	−0.019	76.95
*PtGASA06*	Potri.002G022600.2p	102	10.77	9.03	59.91	0.004	70.95
*PtGASA07*	Potri.002G022700.1p	101	10.57	9.18	48.37	0.007	83.17
*PtGASA08*	Potri.005G239000.2.p	98	10.33	8.00	36.62	0.107	73.82
*PtGASA09*	Potri.005G239100.2.p	102	10.83	8.66	55.48	0.317	80.61
*PtGASA10*	Potri.006G044400.1.p	116	12.72	9.36	57.86	−0.443	58.02
*PtGASA11*	Potri.007G051300.1.p	102	11.17	9.05	47.30	−0.015	67.94
*PtGASA12*	Potri.009G092600.1.p	89	9.95	9.11	34.21	−0.245	55.96
*PtGASA13*	Potri.012G076700.1.p	109	11.93	8.75	36.72	−0.012	77.71
*PtGASA14*	Potri.013G113400.3.p	91	10.04	8.97	49.23	−0.221	60.11
*PtGASA15*	Potri.014G020100.1.p	88	9.65	9.01	27.19	0.003	57.61
*PtGASA16*	Potri.015G071500.1.p	109	11.77	8.88	35.07	0.081	79.45
*PtGASA17*	Potri.017G083000.1.p	107	11.90	9.54	42.59	−0.305	54.67
*PtGASA18*	Potri.017G124200.1.p	95	10.57	9.14	42.43	−0.119	66.74
*PtGASA19*	Potri.019G083900.1.p	110	12.18	9.47	54.87	−0.244	71.91

*M*_W_: molecular weight (kDa), PI: isoelectric point, GRAVY: grand average of hydropathicity.

**Table 2:** Poplar homologous genes.

No.	Gene Name	Homologous Gene	Category	Identify (%)
1	*PtGASA01*	*PtGASA10*	I	75
2	*PtGASA02*	*PtGASA15*	II	73.86
3	*PtGASA02*	*PtGASA12*	II	96.55
4	*PtGASA05*	*PtGASA06*	III	65.05
5	*PtGASA05*	*PtGASA07*	III	78.22
6	*PtGASA05*	*PtGASA09*	III	65
7	*PtGASA06*	*PtGASA09*	III	90.20
8	*PtGASA06*	*PtGASA07*	III	73.74
9	*PtGASA07*	*PtGASA09*	III	74
10	*PtGASA12*	*PtGASA15*	III	74.16
11	*PtGASA13*	*PtGASA16*	III	91.74

## Data Availability

Not applicable.

## Supplementary Materials

The following are available online at https://www.mdpi.com/article/10.3390/ijms23031507/s1.
