# Supplementary material for: Identification and Expression Analysis of the Populus trichocarpa GASA-Gene Family
Source: Int J Mol Sci. 2022 Jan 28;23(3):1507. doi: 10.3390/ijms23031507 (PMC8835824; doi:10.3390/ijms23031507)
Supplement: Supplementary file 1 [file ijms-23-01507-s001.zip › Table S1.pdf]

Table S1. Orthologues of *PtGASA* genes in *Arabidopsis* and apple.

**No.**	Gene name	Homologous gene	Identify(%)
1	*PtGASA06*	*AtGASA01*	72.97
2	*PtGASA06*	*AtGASA11*	67.11
3	*PtGASA07*	*AtGASA01*	66.34
4	*PtGASA08*	*AtGASA11*	66.33
5	*PtGASA09*	*AtGASA01*	72.97
6	*PtGASA12*	*AtGASA10*	65
7	*PtGASA15*	*AtGASA10*	65.17
8	*PtGASA17*	*AtGASA04*	67.59
9	*PtGASA18*	*AtGASA05*	65
1	*PtGASA01*	*MdGASA02*	79.44
2	*PtGASA01*	*MdGASA18*	67.57
3	*PtGASA02*	*MdGASA04*	73.33
4	*PtGASA02*	*MdGASA19*	67.05
5	*PtGASA02*	*MdGASA06*	65
6	*PtGASA05*	*MdGASA05*	67.65
7	*PtGASA06*	*MdGASA05*	69.61
8	*PtGASA06*	*MdGASA15*	76.83
9	*PtGASA07*	*MdGASA05*	67.65
10	*PtGASA09*	*MdGASA05*	67.65
11	*PtGASA09*	*MdGASA15*	74.39
12	*PtGASA10*	*MdGASA02*	65
13	*PtGASA10*	*MdGASA18*	65
14	*PtGASA12*	*MdGASA04*	73.03
15	*PtGASA12*	*MdGASA06*	67.82
16	*PtGASA12*	*MdGASA19*	65.52
17	*PtGASA15*	*MdGASA01*	73.86
18	*PtGASA15*	*MdGASA04*	67.07
19	*PtGASA15*	*MdGASA06*	75
20	*PtGASA15*	*MdGASA07*	75
21	*PtGASA15*	*MdGASA19*	67.82
22	*PtGASA17*	*MdGASA11*	81.31
23	*PtGASA17*	*MdGASA25*	79.44
24	*PtGASA18*	*MdGASA13*	73.68
25	*PtGASA18*	*MdGASA26*	72.37
