# Supplementary material for: Identification and Expression Analysis of the Populus trichocarpa GASA-Gene Family
Source: Int J Mol Sci. 2022 Jan 28;23(3):1507. doi: 10.3390/ijms23031507 (PMC8835824; doi:10.3390/ijms23031507)
Supplement: Supplementary file 1 [file ijms-23-01507-s001.zip › Table S2.pdf]

Table S2. *PtGASA* genes primer.

Gene ID	Forward primer (Reverse primer)	Primer Sequence
PtGASA01	F	GGGCCACTCAAGGCAGTCTTC
PtGASA01	R	CGGGAGGCACACACAAGCAC
PtGASA02	F	ATACGAGGTGCGCAAACGCT
PtGASA02	R	AGGGCACTCGTGCTTGTTCC
PtGASA03	F	GATTGCCCAGAAGCCTGCGA
PtGASA03	R	GGCACTTGGGTTTGCCCTCTT
PtGASA04	F	CATCTCCAGCACCGCCAGTC
PtGASA04	R	GCTTCACGGGAGGCACAGG
PtGASA05	F	GCAAGTCCAGCTGCAGGTTCT
PtGASA05	R	GGTGGCATAGCAAGGGCAAGT
PtGASA06	F	CACGCCTGTGCAAGAGAGCA
PtGASA06	R	TCTGCCGCCATGGGTAGTCA
PtGASA07	F	ATCGACTGTGGTGGCGCTTG
PtGASA07	R	ACACTTGCGTCTGCCTCCAC
PtGASA08	F	CACCGAGCGTGTGGTACCTG
PtGASA08	R	TGCTGGCCATGGTGAGTTGTC
PtGASA09	F	CGAGGCGAGGTGCCAGTTATC
PtGASA09	R	CCGTTCCTGGAGGCACACAG
PtGASA10	F	AAGAGTGCGGACCTCGTTGC
PtGASA10	R	GGCACACACAAGCACTTGGC
PtGASA11	F	GTGCAAGGCTAGGTGTGCCA
PtGASA11	R	GGGCATTTGGAGGTGCCCTT
PtGASA12	F	GCGCAAACGCTGGCATACAG
PtGASA12	R	GGGCACTCATGCTTGTTCCCA
PtGASA13	F	TGCAAGCAGAGATGCAGTCTTCAC
PtGASA13	R	CCTGAGGTCCCAGGAGGCA
PtGASA14	F	GCATAAGGGCTTGCAACACATGC
PtGASA14	R	GCATAGCAAGGGCAGGTATCTTCG
PtGASA15	F	AGTGCTCAGTGAGGTGCTCCA
PtGASA15	R	AGGGCACTCGTGCTTGTTCC
PtGASA16	F	GTGGAAACAGGAGGCTCATGCAA
PtGASA16	R	GCGGCACACACTTGCACCTT
PtGASA17	F	TGGACCTGGGAGTCTCAAGAGC
PtGASA17	R	ACGCAGAGGCACTTCTTGCAG
PtGASA18	F	CCGCTGCTCAGCAACATCACA
PtGASA18	R	AGGGCATGTTTCCTTGTTGCCAT
PtGASA19	F	GCTGCAACAGATGCCGGTGT
PtGASA19	R	CCATGGGTCCTAAGGCTTGCATAACAT
