# Supplementary material for: Identification and Expression Analysis of the Populus trichocarpa GASA-Gene Family
Source: Int J Mol Sci. 2022 Jan 28;23(3):1507. doi: 10.3390/ijms23031507 (PMC8835824; doi:10.3390/ijms23031507)
Supplement: Supplementary file 1 [file ijms-23-01507-s001.zip › Table S3.pdf]

Table S3. Prediction of subcellular localization of PtGASA gene.

Accession number	Predicted location(s)
PtGASA01	Golgi apparatus.
PtGASA02	Cell membrane. Golgi apparatus. Nucleus.
PtGASA03	Golgi apparatus.
PtGASA04	Cell wall.
PtGASA05	Cell membrane. Cell wall. Golgi apparatus. Nucleus.
PtGASA06	Cell wall. Golgi apparatus.
PtGASA07	Cell wall.
PtGASA08	Cell wall. Golgi apparatus.
PtGASA09	Cell membrane. Golgi apparatus. Nucleus.
PtGASA10	Cell wall.
PtGASA11	Golgi apparatus.
PtGASA12	Golgi apparatus. Nucleus.
PtGASA13	Nucleus.
PtGASA14	Golgi apparatus. Nucleus.
PtGASA15	Golgi apparatus.
PtGASA16	Nucleus.
PtGASA17	Golgi apparatus.
PtGASA18	Golgi apparatus. Nucleus.
PtGASA19	Golgi apparatus. Nucleus.
