# Supplementary figures and images for: Identification and Expression Analysis of the Populus trichocarpa GASA-Gene Family
Source: Int J Mol Sci. 2022 Jan 28;23(3):1507. doi: 10.3390/ijms23031507 (PMC8835824; doi:10.3390/ijms23031507)

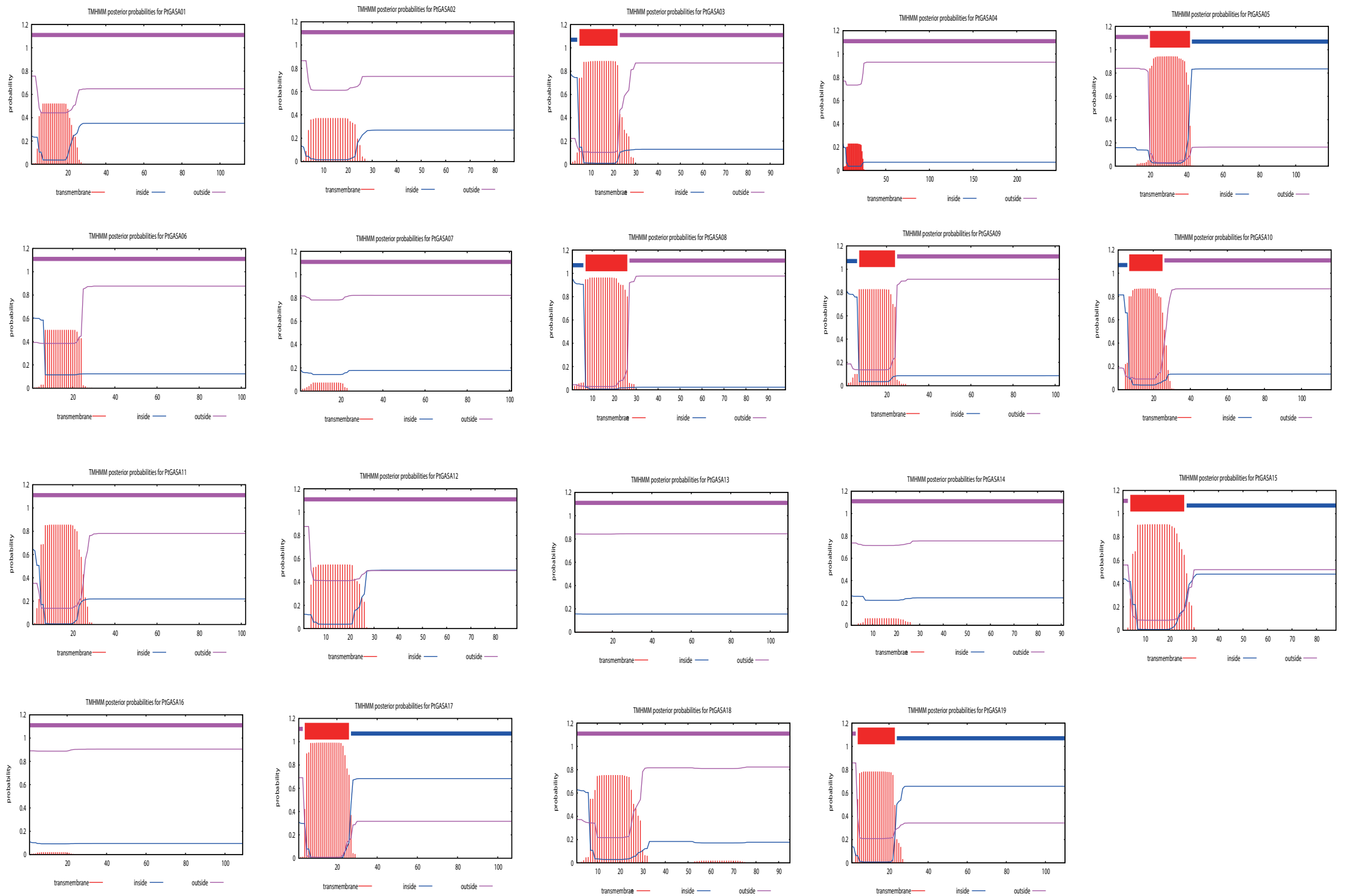

Figure S1. Analysis of the transmembrane region of *PiGASA* proteins

Supplement: Supplementary file 1 [file ijms-23-01507-s001.zip › Figure S1.pdf]

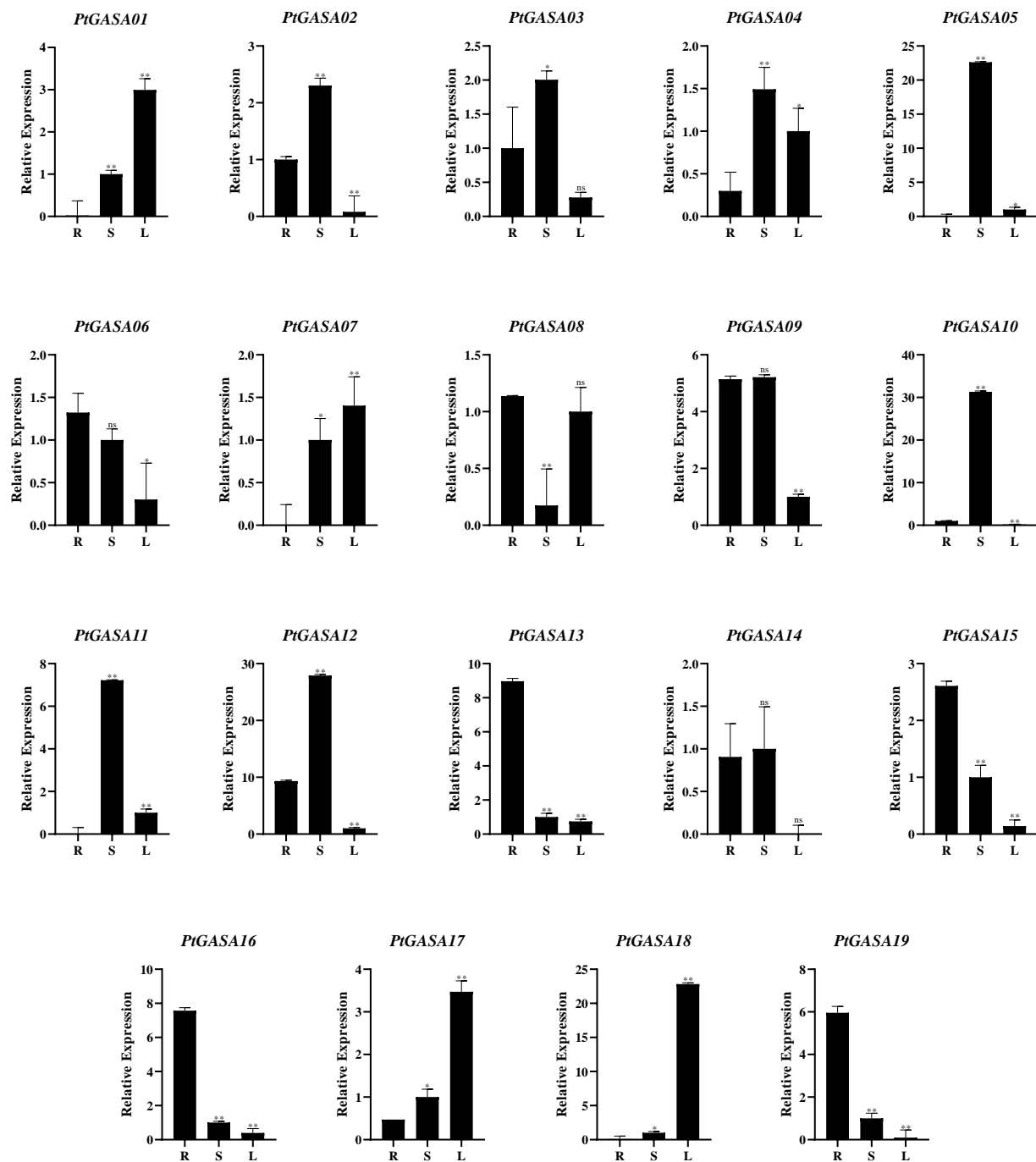

**Figure S2.** Analysis of *PtGASA* expression levels in different tissues (roots, stems, and leaves).

Supplement: Supplementary file 1 [file ijms-23-01507-s001.zip › Figure S2.pdf]
